# The Incidence of Cancers in Patients With Nonfunctional Adrenal Tumors: A Swedish Population-Based National Cohort Study

**DOI:** 10.1210/jendso/bvae154

**Published:** 2024-09-13

**Authors:** Jekaterina Patrova, Buster Mannheimer, Martin Larsson, Jonatan D Lindh, Henrik Falhammar

**Affiliations:** Department of Clinical Science and Education, Södersjukhuset, Karolinska Institutet, 118 83 Stockholm, Sweden; Department of Endocrinology, Södersjukhuset, 118 83 Stockholm, Sweden; Department of Clinical Science and Education, Södersjukhuset, Karolinska Institutet, 118 83 Stockholm, Sweden; Department of Endocrinology, Södersjukhuset, 118 83 Stockholm, Sweden; Department of Molecular Medicine and Surgery, Karolinska Institutet, 171 76 Stockholm, Sweden; Department of Endocrinology, Karolinska University Hospital, 171 76 Stockholm, Sweden; Department of Laboratory Medicine, Division of Clinical Pharmacology, Karolinska Institutet, 141 86 Stockholm, Sweden; Department of Molecular Medicine and Surgery, Karolinska Institutet, 171 76 Stockholm, Sweden; Department of Endocrinology, Karolinska University Hospital, 171 76 Stockholm, Sweden

**Keywords:** malignancy, adrenal incidentaloma, frequency

## Abstract

**Context:**

It is unclear if nonfunctional adrenal tumors (NFAT) are associated with higher cancer incidence.

**Objective:**

To analyze the cancer incidence in patients with NFAT.

**Methods:**

In this national register-based retrospective cohort study, consecutive patients with NFAT identified in Sweden 2005-2019 and matched control individuals without adrenal tumors were followed up to 15 years. Outcome data were collected from national registers and adjusted for confounders. Both cases and controls were followed until newly diagnosed malignancy, death, or until 2019. Individuals with adrenal hormonal excess or prior malignancy were excluded.

**Results:**

Among 17 726 cases, 10 777 (60.8%) were women, and the median age was 65 (IQR, 57-73) years. Among 124 366 controls, 69 514 (55.9%) were women, and the median age was 66 (IQR, 58-73) years. The incidence of any cancer was higher in patients with NFAT compared to controls (hazard ratio [HR] 1.35 95% CI 1.29-1.40; adjusted HR 1.31, 95% CI 1.26-1.37). NFAT was associated with a higher incidence of adrenal, thyroid, lung, stomach and small intestine, kidney, pancreatic, breast, and colorectal cancer. Sensitivity analyses did not change the overall results, but associations were not significantly increased after adjustment in patients with NFAT and appendicitis or gallbladder/biliary tract/pancreas disorders. Cancer incidence may have been underestimated by adjusting for unclear and benign tumors.

**Conclusion:**

The incidence of cancer was increased in patients with NFAT. Long-term follow-up may be indicated.

The frequent use of high-resolution abdominal imaging, such as computed tomography (CT) and magnetic resonance imaging (MRI) scans, has caused an increase in the accidental findings of adrenal tumors (ATs) [1-3]. Earlier research and postmortem investigations suggest that the prevalence of ATs ranges from 1% to 10%, with higher rates observed in older individuals [4].

The manifestation of ATs may involve the overproduction of adrenal hormones, leading to distinct clinical signs. Conversely, a more prevalent scenario is the incidental discovery of ATs through radiological imaging procedures conducted for unrelated diagnostic purposes. Such tumors are called adrenal incidentalomas (AIs) [5].

Most ATs represent benign lesions that are commonly believed to be hormonally inactive, so-called *nonfunctional adrenal tumors* (NFATs). However, mild autonomous cortisol secretion (MACS) [5], that is, overproduction of small quantities of cortisol without overt Cushing syndrome (previously known as subclinical Cushing syndrome) affects 43% to 45% of all AT without clinically apparent hormone excess [6-8].

The most recent European guidelines classify MACS as a serum cortisol concentrations exceeding 50 nmol/L (> 1.8 μg/dL) after a 1-mg dexamethasone suppression test (DST) in the absence of clinical features of overt Cushing syndrome [5]. MACS has been associated with increased cardiometabolic risk [9] and increased morbidity and mortality [10-12]. Recent research indicates that individuals with NFAT also are at increased risk of mortality, primarily due to cardiovascular and cancer-related mortality [13]. However, studies addressing cancer incidence among individuals with NFAT are conspicuously lacking.

The aim of this study was to analyze the cancer incidence in patients with NFAT and compare it with that of control individuals without ATs.

## Methods

### Study Design and Setting

In this population-based retrospective cohort study, the Swedish personal identity number served as the means for linking data across several national registers. The National Patient Register, encompassing data for all hospital inpatient and specialized outpatient services, was used to identify individuals with a first-ever ICD-10 (*International Classification of Diseases, tenth revision*) code of D44.1 (neoplasm of uncertain behavior of the adrenal gland) and/or D35.0 (benign neoplasm of the adrenal gland) spanning the period from January 1, 2005, to December 31, 2019. The Cause of Death Register was used to identify patients who had deceased between 2005 and 2019. Control individuals were selected at random from the Total Population Register and subsequently matched according to age, sex, and municipality at a ratio of 4 controls per case. To explore potential additional confounders (Table 1), we incorporated the longitudinal integrated database for health insurance and labor market studies (LISA), offering detailed socioeconomic information at the individual level. Individuals with a prior diagnosis of any malignancy (as specified by any ICD-10 C code) from 1997 up to 3 months after the first diagnosis of D44.1 or D35.0 were excluded. Individuals diagnosed with hormonal disorders, including Cushing syndrome (ICD-10 code E24), congenital adrenal hyperplasia (ICD-10 code E25) [14], primary aldosteronism (ICD-10 code E26), and pheochromocytoma (ICD-10 code E27.5) from 1997 to 2019 were omitted from both study groups. Our aim in excluding all hormonally active lesions was to narrow the focus of the study to solely NFAT.

**Table 1.** Primary and secondary outcomes, potential confounders, and other conditions based on ICD diagnoses from the national patient registry including both inpatient and outpatient care

	ICD-10 code
**Primary outcome**	
Any cancer	Any C code
**Secondary outcomes**	
Adrenal cancer	C74
Lung cancer	C34
Pancreatic cancer	C25
Colorectal cancer	C18, C20
Prostate cancer	C61
Breast cancer	C50
Ovarian cancer	C56.9, C79.6
Uterine cancer	C55, C54.9
Melanoma	C43
Kidney cancer	C64, C65
Bladder cancer	C66, C67
Stomach and small intestine cancer	C16, C17
Thyroid cancer	C73
Brain cancer	C71
Lymphoma	C81-C86
Other cancer sites	C49
**Potential Confounders**	
Age	
Sex	
Obesity	E66
Cancer in situ	D00-D09
Benign neoplasms	D10-D36 (excluding D35.0)
Neoplasms of uncertain behavior	D347-D48 (excluding D44.1)
Pancreas diseases	K85, K860-1
Inflammatory bowel disease	K51, K50
Liver diseases	K70-K77
Alcohol misuse	E24.4, F10, G62.1, G72.1, I42.6, K29.2, K70, K86
Socioeconomic factors	Education:
	Primary and secondary (< 9 years)
	Primary and secondary (9-10 years)
	Upper secondary (< 2 years)
	Upper secondary (3 years)
	Postsecondary (< 3 years)
	Postsecondary (≥ 3 years)
	Postgraduate education
	Income (Disposable income for one person in SEK)
	Unemployment (Number of days in unemployment)
**Other interventions/diagnosis**	
Acute appendicitis	K35
Gallbladder/biliary tract/pancreas diseases	K80-87

The index date was set at 3 months after the initial D44.1 or D35.0 diagnosis to reduce potential detection bias. This accounts for cases where patients had undergone radiological imaging for suspected malignancy, and the diagnosis of NFAT followed shortly after the initial imaging but before the malignancy was diagnosed. Sensitivity analyses were performed in subgroups of individuals in whom we would presume controls would also have had a CT: those with acute appendicitis (in whom we would also assume there was no concern of cancer, ICD-10 code K35), and in patients with a combination of gallbladder/biliary tract/pancreas disorders (ICD-10 codes K80-K87). Cases were included if they had received an appendicitis or gallbladder/biliary tract/pancreas diagnosis within 6 months prior to the D44.1 and/or D35.0 diagnosis, while the index date for controls was determined based on the date of their first-ever (since 1997) appendicitis or gallbladder/biliary tract/pancreas diagnosis. In 2 additional sensitivity analyses, the index date was shifted forward 6 and 12 months, respectively, after the initial D44.1 or D35.0 diagnosis to further prevent situations in which patients had undergone imaging for suspected malignancy that was then confirmed shortly after the imaging that found the NFAT. This cohort or part of it has been used in previous studies [13-16].

### Statistical Analysis

Descriptive statistics included percentages, means, medians and interquartile ranges (IQR) as appropriate. Cancer incidences during the follow-up were illustrated through Kaplan-Meier curves. We assessed the incidence of any cancer (primary outcome) as well as specific cancers (secondary outcomes) by comparing cases and controls. We utilized Cox proportional hazard ratios (HRs) with 95% CIs, both with and without adjustment for potential confounders (Table 1). Furthermore, analyses were repeated within subgroups based on sex and age. If no case or control had a specified cancer, Cox proportional HR with 95% CI could not be calculated, and log rank test was used instead. *P* values < .05 were considered statistically significant. Statistical analysis was conducted in R, version 4.0.3 (Vienna, Austria. URL: https://www.R-project.org/)

## Results

### Study Population

A total of 17 726 patients with NFAT and 124 366 control individuals were included. Of those with NFAT, 10 777 (60.8%) were female and 6949 (39.2%) were male. The median age at the time of the NFAT diagnosis was 65 years (IQR, 57-73). The most common comorbidities among both cases and controls were previous benign or unclear tumors (excluding D35.0 and D44.1), diabetes mellitus and chronic obstructive pulmonary disease (COPD). Table 2 presents a selection of medical conditions and socioeconomic factors in the study population at the index date.

**Table 2.** Medical characteristics and socioeconomic factors among cases with nonfunctioning adrenal tumors and controls at baseline

	Number of cases (%) (n = 17 726)	Number of controls (%) (n = 124 366)
**Sex**		
Women	10 777 (60.8)	69 514 (55.9)
Men	6949 (39.2)	54 852 (44.1)
Median age (IQR), years	65 (57-73)	66 (58-73)
**Diagnosis**		
Ca in situ	227 (1.3)	1 138 (0.9)
Benign tumor*^a^*	4 080 (23)	15 993 (12.9)
Unclear tumor*^a^*	2 509 (14.2)	6 815 (5.5)
Pancreas diseases	445 (2.5)	938 (0.8)
Inflammatory bowel disease	406 (2.3)	1 389 (1.1)
Liver	432 (2.4)	1000 (0.8)
COPD	1 726 (9.7)	3417 (2.7)
Diabetes mellitus	2 966 (16.7)	9 706 (7.8)
Alcohol	909 (5.1)	3 231 (2.6)
Obesity*^b^*	1300 (7.3)	2 786 (2.2)
Appendicitis recent	155 (0.9)	2 154 (1.7)
Gallbladder/biliary tract/pancreas recent	802 (4.5)	10 213 (8.2)
**Socioeconomic factors**		
Education		
Primary and secondary (< 9 years)	3411 (19.2)	22 780 (18.3)
Primary and secondary (9-10 years)	2064 (11.6)	11 528 (9.3)
Upper secondary (< 2 years)	5918 (33.4)	36 133 (29)
Upper secondary (3 years)	2007 (11.3)	15 255 (12.3)
Postsecondary (< 3 years)	1638 (9.2)	14 141 (11.4)
Postsecondary (≥ 3 years)	1956 (11)	18 344 (14.6)
Postgraduate	98 (0.6)	1242 (1)
Missing (NA)	634 (3.6)	4943 (4.0)
Annual income (SEK)	164 600 (127 300-242 100)	179 800 (130 500- 269 100)
Unemployment days	0 (0; 0)	0 (0; 0)

Abbreviations: COPD, chronic obstructive pulmonary disease; IQR, interquartile range; SEK, Swedish krona.

^*a*^Excluding benign adrenal tumors and unclear adrenal tumors.

^*b*^Please note this only includes those who have received an ICD-10 code of obesity, which is a highly selected group.

### The Incidence of Any Cancer

**Figure 1.**
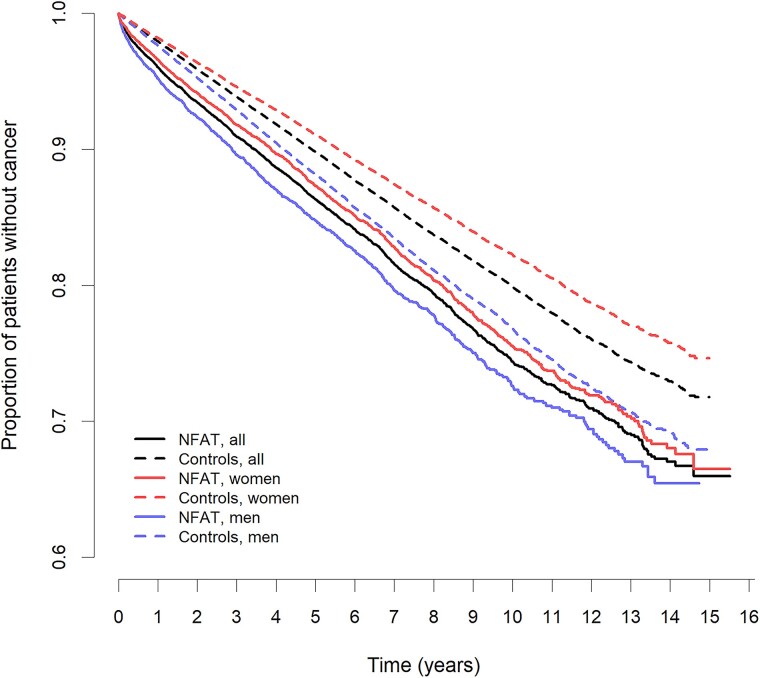
Kaplan-Meier curves with 95% CIs illustrating the incidence of any cancer in 17 726 individuals with nonfunctional adrenal tumors compared to 124 366 controls (both sexes); 10 777 female patients with nonfunctional adrenal tumors compared to 69 514 female controls; 6949 male patients with nonfunctional adrenal tumors compared to 54 852 male controls. Cox proportional hazard ratio analysis, adjusted for potential confounders, all *P* values between patients and controls are < .001.

In total, 2717 (15.3%) cases and 15 015 (12.1%) controls were diagnosed with cancer during a follow-up of up to 15 years (median 5.0 years, IQR 2.3-8.2). Cancer was diagnosed more often in individuals with NFAT than in controls (HR 1.35 [95% CI 1.29-1.40]; adjusted HR [aHR] 1.31 [95% CI 1.26-1.37]). This association was evident in both sexes with slightly higher incidence in female individuals (Table 3, Fig. 1).

**Table 3.** Hazard ratios for specific cancers in individuals with nonfunctional adrenal tumors, as well as in sex groups, compared to controls

	All				Females				Males			
	(n = 17 726)				(n = 10 777)				(n = 6949)			
	HR (95% CI)	*P* value	aHR (95% CI)	*P* value	HR (95% CI)	*P* value	aHR (95% CI)	*P* value	HR (95% CI)	*P* value	aHR (95% CI)	*P* value
Any cancer	1.35 (1.29-1.40)	< .001	1.31 (1.26-1.37)	< .001	1.44 (1.36-1.52)	< .001	1.34 (1.26-1.41)	< .001	1.28 (1.20-1.36)	< .001	1.29 (1.21-1.37)	< .001
Adrenal cancer*^a^*	NA	NA	NA	NA	NA	NA	NA	NA	NA	NA	NA	NA
Thyroid cancer	3.45 (2.32-5.13)	< .001	2.89 (1.89-4.41)	< .001	3.16 (1.95-5.13)	< .001	2.72 (1.62-4.54)	< .001	3.95 (1.98-7.86)	< .001	3.44 (1.65-7.14)	< .001
Lung cancer	2.81 (2.51-3.16)	< .001	2.30 (2.04-2.60)	< .001	3.12 (2.69-3.61)	< .001	2.48 (2.12-2.90)	< .001	2.45 (2.04-2.95)	< .001	2.09 (1.72-2.54)	< .001
Stomach and small intestine cancer	2.16 (1.70-2.76)	< .001	1.93 (1.49-2.50)	< .001	1.50 (1.02-2.20)	.04	1.31 (0.87-1.95)	.19	3.06 (2.22-4.20)	< .001	2.71 (1.93-3.79)	< .001
Kidney cancer	2.11 (1.69-2.63)	< .001	1.74 (1.38-2.20)	< .001	2.11 (1.52-2.93)	< .001	1.42 (1.00-2.02)	.05	2.23 (1.65-3.00)	< .001	2.06 (1.51-2.81)	< .001
Bladder cancer	1.74 (1.47-2.06)	< .001	1.76 (1.47-2.11)	< .001	2.14 (1.58-2.89)	< .001	1.89 (1.37-2.59)	< .001	1.73 (1.41-2.13)	< .001	1.70 (1.37-2.10)	< .001
Pancreatic cancer	1.71 (1.39-2.10)	< .001	1.49 (1.20-1.85)	< .001	1.85 (1.42-2.41)	< .001	1.56 (1.18-2.06)	.002	1.51 (1.08-2.12)	.02	1.38 (0.97-1.96)	.07
Breast cancer	1.32 (1.16-1.50)	< .001	1.18 (1.03-1.35)	.01	1.21 (1.06-1.37)	.004	1.17 (1.02-1.34)	.02	2.89 (1.14-7.33)	.03	2.17 (0.86-5.52)	.10
Colorectal cancer	1.18 (1.03-1.36)	.02	1.19 (1.03-1.38)	.02	1.40 (1.17-1.67)	< .001	1.38 (1.15-1.66)	< .001	0.91 (0.71-1.16)	.46	0.95 (0.74-1.22)	.68
Prostate cancer	0.85 (0.75-0.95)	.007	1.04 (0.91-1.17)	.59	NA	NA	NA	NA	0.95 (0.84-1.08)	.43	1.04 (0.91-1.17)	.57
Ovarian cancer	2.33 (1.00-5.46)	.05	2.03 (0.87-4.76)	.10	1.84 (0.75-4.54)	.19	1.72 (0.70-4.25)	.24	NA	NA	NA	NA
Uterine cancer	0.58 (0.21-1.59)	.29	0.50 (0.18-1.39)	.18	0.53 (0.19-1.47)	.22	0.50 (0.18-1.39)	.18	NA	NA	NA	NA
Melanoma	0.94 (0.76-1.16)	.56	0.95 (0.76-1.18)	.63	0.85 (0.63-1.14)	.28	0.83 (0.61-1.13)	.23	1.08 (0.80-1.45)	.63	1.10 (0.81-1.49)	.54
Brain cancer	1.10 (0.76-1.61)	.6	1.04 (0.70-1.54)	.83	1.09 (0.64-1.84)	.76	1.00 (0.57-1.73)	.99	1.17 (0.68-2.00)	.58	1.07 (0.61-1.86)	.83
Lymphoma	1.25 (0.10-1.61)	.07	1.27 (0.98-1.64)	.07	1.28 (0.91-1.79)	.15	1.22 (0.86-1.73)	.27	1.26 (0.88-1.82)	.21	1.31 (0.90-1.92)	.16
Other cancer sites	1.70 (1.16-2.48)	.006	1.52 (1.03-2.26)	.04	2.72 (1.73-4.29)	< .001	2.43 (1.50-3.92)	< .001	0.74 (0.34-1.61)	.45	0.63 (0.29-1.40)	.26

Abbreviations: aHR, adjusted hazard ratio; HR, hazard ratio; NA, not applicable.

^*a*^Since no control subject developed any adrenal cancer, calculating HR with 95% CI was not meaningful. However, using log rank test, NFAT was associated with an increased risk of adrenal cancer in all comparisons with controls (*P* < .001).

### The Incidence of Specific Types of Cancer

The incidence of most investigated specific cancers was significantly higher among patients with NFAT compared to controls without adrenal tumors. These were adrenal, thyroid, lung, stomach and small intestine, kidney, bladder, pancreatic, breast, colorectal, and other cancers (Table 3).

### Age-Related Incidence of Any Type and Specific Types of Cancer

The cancer incidence was slightly higher in patients with NFAT below 65 years of age (especially thyroid and kidney cancers), but overall cancer incidence among individuals with NFAT did not vary markedly between age groups (Supplementary Table S1, Supplementary Fig. S1) [17], nor the specific types of cancer (Table 3, Supplementary Table S1) [17].

### Adrenalectomy

**Figure 2.**
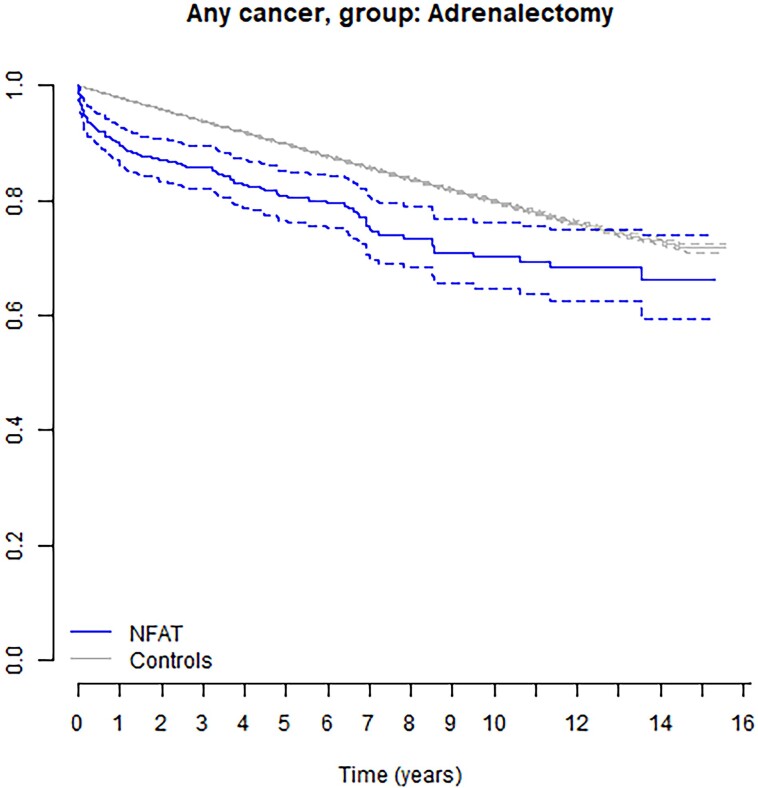
Kaplan-Meier curve with 95% CIs illustrating cancer incidence among 593 individuals with nonfunctional adrenal tumors who had had adrenalectomy within 1 year after diagnosis, compared to 121 247 controls. Cox proportional hazard ratio analysis, adjusted for potential confounders, *P* value is < .001.

Out of the total 17 726 individuals diagnosed with a NFAT, 593 patients (3.4%), including 356 females (60.0%), underwent an adrenalectomy within 1 year after the NFAT diagnosis. Following the adrenalectomy, the overall cancer prevalence (HR 1.22 [95% CI 1.00-1.49], *P* value = .05; aHR 1.40 [95% CI 1.15-1.71], *P* < .001) remained elevated compared to the control group (Fig. 2).

### Sensitivity Analyses

Among individuals with acute appendicitis or gallbladder/biliary tract/pancreas diseases, the incidence of cancer remained significantly increased in those with NFAT compared to controls, albeit not significantly so after adjustments (appendicitis: HR 1.76 [95% CI 1.14-2.72], *P* value = .01, aHR 1.44 [95% CI 0.92-2.24], *P* value = .11; gallbladder/biliary tract/pancreas disorder: HR 1.48 [95% CI 1.24-1.77], *P* value < .001, aHR 1.09 [95% CI 0.89-1.35], *P* value = .40). The increased cancer incidence in patients with NFAT compared with controls remained when the index date was shifted 6 months (HR 1.28 [95% CI 1.22-1.33], *P* value < .001, aHR 1.24 [95% CI 1.19-1.30], *P* value < .001) or 12 months after the NFAT diagnosis (HR 1.25 [95% CI 1.19-1.31], *P* value < .001, aHR 1.21 [95% CI 1.15-1.27], *P* value < .001).

## Discussion

This large population-based study, involving 17 726 individuals with NFAT and 124 366 control individuals without adrenal lesions, examined the comparative cancer incidence. Cancer incidence was found to be increased in patients with NFAT, with a slightly larger increase observed among female individuals. The finding of increased cancer incidence remained in patients with NFAT irrespective of age, adrenalectomy, or in the different sensitivity analyses.

The predominant increase in cancer incidence involved adrenal, thyroid, lung, stomach and small intestine, kidney, bladder, pancreatic, breast, and colorectal cancers. The cancer incidence was particularly high in female patients with NFAT or in those patients with NFAT less than 65 years of age (especially thyroid and kidney cancers). However, breast and colorectal cancers were only associated with slight increases in incidence (breast cancer aHR 1.18, colorectal cancer aHR 1.19) that may not be clinically concerning.

Although neoplastic metastases are the most common malignancies in the adrenal glands [18], few studies have investigated the incidence of nonadrenal cancers among ATs. This excludes studies of pheochromocytomas or adrenocortical cancers where familial syndromes associated with different cancers are common [19, 20]. Yener et al conducted a study involving 29 patients with AI and nonadrenal malignancies, comparing them with a control group of 110 patients with AI but without malignancies [21]. After a 24-month follow-up period, there were no significant differences observed in the size and hormonal activity of AIs between the groups [21]. These results align with the results reported by Tsvetov et al, who demonstrated that among patients with AI and prior cancer diagnosis, after 24 months of follow-up, tumor progression and functional abnormalities showed similar patterns regardless of the presence or absence of concurrent cancer in AI patients [22].

Kawate et al found a higher prevalence of nonadrenal malignancies among patients with bilateral AIs (BAIs) and MACS [23]. The study population was comparatively small (n = 27). Of the 11 patients diagnosed with BAI and MACS 8 (73%) manifested additional malignancies in different tissues, including breast, thyroid, colon, lung, basal cell, bladder, endometrium, and ureter carcinoma [23]. Four of the 8 patients with nonadrenal malignancies were diagnosed with primary bilateral macronodular adrenocortical hyperplasia (PBMAH), while the remaining patients had adenomas [23]. Interestingly, patients with both unilateral and bilateral AIs were similarly exposed to MACS; however, the prevalence of nonadrenal malignancy was significantly higher in patients with BAIs compared to those with unilateral AIs [23]. This observation led to the speculation that genetic alterations in tissues of patients with BAIs may have a greater impact on tumorigenesis than exposure to subtle hypercortisolemia.

Several independent researchers have observed the diffuse, nonmetastatic enlargement of adrenal glands among patients with nonadrenal malignancies, particularly among those who had high post-DST cortisol levels [24, 25]. It is also known that chronic stress might be a common factor, as it can lead to both adrenal hyperplasia and alterations in the immune system, impairing cancer defense mechanisms [26]. Furthermore, prior studies have demonstrated a higher prevalence of metabolic syndrome [27], type 2 diabetes mellitus [1], and increased carotid intima-media thickness [28] among patients with NFAT. These findings indicate that NFAT might potentially cause fluctuating cortisol levels that go undetected by current laboratory tests or a dysregulated cyclic cortisol secretion.

Moreover, earlier studies have shown that age-related and experimentally induced dysfunctions in cortisol feedback mechanisms contribute to increased tumor growth, suppression of apoptosis [29], and initiation of ovarian cancer [30] in animal models.

Notably, in our study, individuals who underwent adrenalectomy within the first year of NFAT diagnosis still had increased cancer incidence. Given that all adrenal carcinomas and functional adrenal tumors were excluded from the outset of the study, all patients who underwent adrenalectomy had NFATs. Therefore, adrenalectomy was presumably performed due to enlarged size and/or higher density of the tumors. This finding supports the hypothesis that genetic disruptions in individuals with NFATs contribute to the development of malignancy.

Furthermore, our study showed a slightly stronger association between NFAT and malignancies of thyroid and kidney origin among individuals younger than 65 years. It is already known that patients with ATs have a higher prevalence of benign thyroid nodules [31-33]. Moreover, others previously reported an increased risk of subsequent cancers, including thyroid and kidney cancer, after detection of an adrenal malignancy [34].

Tumor development typically occurs as a result of abnormal expression of genes related to growth [35]. Therefore, if an AT is present and especially if it grows, it indicates neoplastic potential which could potentially lead to the development of other tumors and increase incidence of any malignancy [11]. Consequently, the underlying risk of tumorigenesis in patients with NFATs is likely elevated compared to control subjects.

We previously published a study based on the same cohort where we observed a higher mortality rate related to malignancies among individuals with NFAT [13]. A total of 851 of 3719 (22.9%) deaths among cases were attributed to cancer compared with 3863 out of 19 343 deaths (20.0%) in controls. After adjustments for age, sex, and other potential confounding factors, a statistically significantly higher cancer-related mortality among patients with NFAT was observed (aHR 1.54 [95% CI 1.42-1.67], *P* < .001) [13]. Kjellbom et al, with a follow-up time similar to our previous study found an even higher cancer mortality (51 of 170 [30%] patients, no controls included) in AI with no difference according to post-DST cortisol concentrations [7]. These studies suggest that cancer mortality in NFAT is increased.

The sensitivity analyses showed mixed results. Moving the index date 6 or 12 months after the NFAT diagnosis did not change the overall results. However, when only analyzing those diagnosed with NFAT just after being diagnosed with acute appendicitis or gallbladder/biliary tract/pancreas disorders and comparing them to controls just diagnosed with the same conditions, the cancer risk remained significantly increased in the unadjusted analyses, but not in the adjusted ones. There are several possible explanations for this discrepancy. The number of recent acute appendicitis or gallbladder/biliary tract/pancreas disorders was small, resulting in a limited power to detect differences between the groups. To clarify this issue, a large study with controls that had also had an imaging similar to patients with NFAT should ideally be conducted, reducing the influence of baseline differences in unknown confounders that could cause spurious associations.

Our study has several limitations. We did not have access to either biochemical results or the radiological reports. Consequently, some proportion of individuals may have been defined as NFAT despite slightly elevated cortisol concentrations. As discussed above, slightly elevated cortisol concentrations could relate to higher cancer incidence. However, it is known that an ICD-10 code of Cushing syndrome overestimates overt Cushing in register-based research and likely numerous patients with an ICD-10 code of Cushing syndrome have in fact MACS diagnosis [36]. Thus, it is likely that many patients with MACS were not included in our NFAT patient group. Furthermore, despite the exclusion of patients with documented primary aldosteronism, the potential presence of individuals with subclinical aldosteronism in the cohort could raise the concerns regarding its impact on cancer incidence [36], given the known association between primary aldosteronism and elevated risk of malignancy [37]. Moreover, it can be assumed that patients having cancer-related symptoms undergo radiological imaging more frequently and even if we tried to account for that by excluding individuals diagnosed with malignancy within the first 3 months (and 6 and 12 months in the sensitivity analyses) after the NFAT diagnosis, this may not have been enough to eliminate the potential bias. Furthermore, although we adjusted for a broad range of potential confounders, such as comorbidities and socioeconomic factors, residual confounding cannot be excluded as discussed above. By adjusting for unclear and benign tumors we may be underestimating the cancer incidence; however, we preferred to have a more conservative approach to the study. Moreover, since most control individuals probably had no prior imaging, some of them may have had an AT, thus underestimating the results.

## Conclusion

The incidence of cancer was increased in patients with NFAT. Long-term follow-up may be indicated, involving history and physical examination to track symptoms and signs over time.

## Data Availability

Restrictions apply to the availability of some or all data generated or analyzed during this study to preserve patient confidentiality or because they were used under license. The corresponding author will on request detail the restrictions and any conditions under which access to some data may be provided.

## Abbreviations

AI: adrenal incidentaloma
AT: adrenal tumor
BAI: bilateral adrenal incidentaloma
CT: computed tomography
DST: dexamethasone suppression test
HR: hazard ratio
IQR: interquartile range
MACS: mild autonomous cortisol secretion
NFAT: nonfunctional adrenal tumor
